# Platelet-rich plasma in peripheral nerve injury repair: a comprehensive review of mechanisms, clinical applications, and therapeutic potential

**DOI:** 10.3389/ebm.2025.10746

**Published:** 2025-09-23

**Authors:** Kai Shang, Yang Liu, Abdul Qadeer

**Affiliations:** ^1^ Department of Orthopedic, The Affiliated Taian City Centeral Hospital of Qingdao University, Taian, Shandong, China; ^2^ Department of Cell Biology, School of Life Sciences, Central South University, Changsha, China

**Keywords:** peripheral nerve injury, platelet-rich plasma, nerve regeneration, growth factors, neurotrophic factors, schwann cells, therapeutic target, regenerative medicine

## Abstract

Peripheral nerve injuries (PNIs) pose a significant clinical challenge, often leading to incomplete functional recovery despite current treatments. Platelet-rich plasma (PRP), which contains high levels of growth factors and bioactive molecules, has emerged as a promising regenerative therapy for nerve repair and restoring function. This review consolidates current evidence on PRP applications in treating peripheral nerve injuries, examining molecular mechanisms, clinical outcomes, and therapeutic potential. PRP markedly enhances nerve regeneration, improves recovery of sensory and motor functions, and alleviates neuropathic pain across various nerve injuries. It promotes axonal growth, reduces scar formation, stimulates Schwann cell proliferation, and modulates inflammation through the release of neurotrophic factors, including PDGF, VEGF, TGF-β, and IGF-1. Combining PRP with surgical techniques and biomaterial scaffolds yields better therapeutic results. Key factors influencing efficacy include platelet concentration, leukocyte content, activation methods, and patient-specific variables. PRP is a safe and effective option for peripheral nerve injury repair. However, challenges persist in standardizing preparation protocols, optimizing treatment timing, and fully understanding molecular mechanisms. Future research should focus on personalized PRP formulations, combination therapies, and large-scale randomized controlled trials to develop definitive clinical guidelines.

## Impact statement

Peripheral nerve injuries often lead to long-term disability, and current treatment options offer limited functional recovery. This review is important because it consolidates and critically evaluates the growing body of research on the use of platelet-rich plasma (PRP) as a novel, biologically based therapy for peripheral nerve repair. While PRP has gained attention in various fields of regenerative medicine, its role in nerve healing is still emerging and not yet standardized. By bringing together recent findings from both preclinical and clinical studies, this work provides new insight into how PRP promotes nerve regeneration through anti-inflammatory effects, stimulation of nerve-supporting cells, and delivery of growth factors that accelerate healing. It also explores how PRP can be combined with existing surgical and biomaterial approaches for improved outcomes. This review contributes to the field by highlighting both the therapeutic promise and the current limitations of PRP, and by outlining future research directions needed to optimize its clinical application. As such, it helps define a clearer path forward for integrating PRP into routine nerve injury management.

## Introduction

The central nervous system (CNS), comprising the brain and spinal cord, acts as the central control hub that communicates with various body organs via an extensive network of nerve fibres extending throughout the peripheral nervous system. This communication occurs through electrical and chemical signals that facilitate coordinated physiological functions and responses to environmental stimuli. These peripheral nerves can be systematically classified based on their anatomical locations and functional characteristics into three primary categories: mixed nerves (containing both sensory and motor fibres), motor nerves (responsible for muscle contraction and movement), and sensory nerves (transmitting sensory information from receptors to the CNS) [1, 2].

Peripheral nerve injury (PNI) represents a significant global health concern and a leading cause of long-term disability, affecting millions of individuals worldwide with substantial socioeconomic implications. The consequences of PNI are often devastating, resulting in severe sensory-motor dysfunction that impairs daily activities, chronic neurogenic pain that significantly reduces quality of life, and potential permanent disability requiring long-term rehabilitation [3–5]. The etiology of PNI is diverse and multifactorial, encompassing neurodegenerative diseases that progressively damage nerve structure and function, acute open trauma from accidents or surgical procedures, and chronic nerve compression syndromes such as carpal tunnel syndrome or cubital tunnel syndrome [6, 7].

In the anatomically complex head and neck region, peripheral nerve injuries pose challenges due to the critical functional roles of affected nerves. Damage commonly affects several key cranial and peripheral nerves, including the inferior alveolar nerve (resulting in altered sensation in the lower lip and chin), the lingual nerve (causing taste disturbances and tongue numbness), the facial nerve (leading to facial paralysis and expression difficulties), and the hypoglossal nerve (affecting tongue movement and speech articulation). These injuries can severely impact essential functions such as mastication, speech, facial expression, and overall oral function [8].

While peripheral nerve fibres demonstrate a remarkable regenerative capacity and can achieve spontaneous healing within weeks to months under optimal conditions, this natural recovery process is often incomplete or insufficient, especially in cases involving significant nerve damage, large gaps, or unfavourable local conditions [9]. The clinical reality presents considerable therapeutic challenges, as fewer than half of patients with documented PNI undergo surgical nerve repair, often due to factors such as delayed diagnosis, patient comorbidities, or lack of specialized surgical expertise. Among those who do receive surgical intervention, only 40–50% attain complete functional recovery, highlighting the limitations of current treatment approaches and the urgent need for improved options [10]. Current management strategies include both surgical techniques (such as direct repair, nerve grafting, and nerve transfers) and conservative methods, with non-surgical treatments encompassing targeted physiotherapy programmes, emerging cell-based therapies using stem cells or Schwann cells (SC), and pharmaceutical interventions aimed at managing pain and encouraging nerve regeneration [3–5, 11].

Platelet-rich plasma (PRP) represents an innovative autologous biological therapeutic derived through centrifugal separation of the patient’s own blood, specifically isolating the plasma fraction enriched with platelet concentrations that typically exceed normal physiological levels by 3-5-fold [12, 13]. This preparation process involves collecting whole blood, followed by specific centrifugation protocols that concentrate platelets while preserving their functional integrity and bioactive properties. The resulting PRP product serves as a potent reservoir of endogenous bioactive molecules, being particularly rich in multiple growth factors and cytokines essential for tissue repair and regeneration. These include granulocyte-macrophage colony-stimulating factor (GM-CSF) for cellular proliferation, vascular endothelial growth factor A (VEGF-A) for angiogenesis, epithelial growth factor (EGF) for cellular differentiation, transforming growth factor β (TGF-β) for tissue remodeling, platelet-derived growth factor (PDGF) for cellular migration and proliferation, hepatocyte growth factor (HGF) for neuroprotection, and insulin-like growth factor 1 (IGF-1) for nerve regeneration [14].

PRP has established a substantial clinical track record demonstrating therapeutic efficacy across diverse medical applications, including accelerated healing in sports-related injuries, enhanced recovery in spinal cord trauma, improved wound healing in chronic conditions, and successful outcomes in plastic and reconstructive surgery procedures [15]. Specifically in the context of peripheral nerve injuries, PRP exhibits multifaceted therapeutic mechanisms, demonstrating significant neurogenic properties that promote nerve fibre regeneration, neuroprotective effects that prevent secondary nerve degeneration, and anti-inflammatory activities that modulate detrimental neuroinflammation while creating a favourable microenvironment for healing [8, 16–18]. These comprehensive therapeutic effects are mediated through PRP’s complex role in orchestrating nerve regeneration processes, including SC proliferation, axonal sprouting, remyelination, and its documented capacity to alleviate debilitating neuropathic pain through modulation of inflammatory pathways and pain signaling mechanisms [19]. Compelling clinical evidence continues to emerge supporting PRP’s therapeutic potential, as exemplified by the case study conducted by García de Cortázar et al. [20], who documented satisfactory neurological recovery and functional improvement in a patient with significant nerve injury following a structured PRP treatment protocol administered over 11 months [20].

Given the demonstrated therapeutic potential of PRP in managing peripheral nerve injuries, along with the urgent clinical need for more effective treatment methods to improve functional recovery, this comprehensive review aims to systematically summarize, critically analyze, and discuss current research progress on PRP applications for PNI. The review will evaluate both preclinical and clinical evidence, treatment protocols, outcomes, and future research directions to enhance PRP-based therapies for peripheral nerve injury management.

## Application of PRP in the treatment of peripheral nerve injury

Accumulating evidence from both preclinical and clinical studies shows that PRP has multiple therapeutic properties vital for peripheral nerve repair. The main reason for PRP’s effectiveness is its ability to regulate neuroinflammation through a dual mechanism involving direct platelet-derived anti-inflammatory mediators and the recruitment of reparative cell populations that release additional anti-inflammatory factors [21–23]. When activated, platelets in PRP release stored anti-inflammatory cytokines such as interleukin-10 (IL-10) and TGF-β, while also attracting macrophages, mesenchymal stem cells, and other regenerative cells to the injury site. These recruited cells further enhance the anti-inflammatory environment by secreting more anti-inflammatory mediators, resulting in a sustained therapeutic effect that lasts beyond the initial platelet activation phase.

Beyond these anti-inflammatory properties, PRP exhibits significant neuroprotective capabilities by preventing secondary neuronal death and promoting axonal survival following peripheral nerve injury. Furthermore, the neurogenic properties of PRP are mediated through the release of neurotrophic factors, including nerve growth factor (NGF), brain-derived neurotrophic factor (BDNF), and neurotrophin-3 (NT-3), which stimulate axonal sprouting, guide nerve fiber growth, and support the maintenance of neuronal phenotype during the regeneration process [1, 24]. Consequently, these growth factors work synergistically to create an optimal microenvironment that facilitates both proximal nerve stump survival and distal target reinnervation.

Building upon these fundamental mechanisms, the therapeutic potential of PRP has been extensively investigated across various peripheral nerve injuries, with consistent positive outcomes reported for multiple anatomical locations. Comprehensive studies have documented PRP’s efficacy in treating injuries to major peripheral nerves, including the sciatic nerve, facial nerve, median nerve, and even applications extending to central nervous system pathologies [1, 21–24]. Moreover, the versatility of PRP treatment is further evidenced by its successful application in diverse clinical scenarios, ranging from complete nerve transection repairs to functional restoration across peripheral nerve gaps [25].

In addition to its regenerative capabilities, PRP therapy has demonstrated significant analgesic properties in treating neuropathic pain associated with peripheral nerve injuries. The pain-relieving mechanisms involve the downregulation of pro-inflammatory cytokines and the modulation of pain signaling pathways at both peripheral and central levels [18, 25–27]. Notably, recent studies have shown that PRP application effectively reduces neuropathic pain in osteoarthritis patients by specifically downregulating microglial activation in the spinal cord, thereby interrupting the central sensitization processes that contribute to chronic pain states [28]. This dual peripheral and central mechanism of pain relief represents a significant advantage over conventional analgesic approaches.

The mechanisms underlying these regenerative effects are significantly mediated through PRP’s impact on Schwann cell biology. SC play a crucial role in peripheral nerve regeneration by providing structural support, producing neurotrophic factors, and facilitating remyelination of regenerating axons. Supporting this understanding, Salarinia et al. [29] demonstrated that PRP treatment significantly enhances SC proliferation in experimental spinal cord injury models in rats, leading to improved functional outcomes. Subsequently, investigations have confirmed PRP’s ability to promote both SC migration to injury sites and their subsequent proliferation, creating a cellular environment conducive to nerve repair [25, 30]. These cellular effects are attributed to the growth factors present in PRP, particularly PDGF and HGF, which specifically target SC receptors and activate proliferation pathways.

**FIGURE 1:**
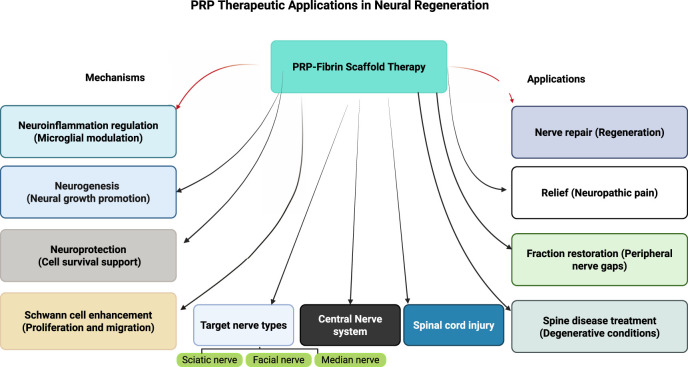
Comprehensive framework of platelet-rich plasma therapeutic applications in neural regeneration.

Collectively, the diverse applications and mechanisms of PRP in peripheral nerve regeneration have been systematically documented across multiple research studies, with findings consistently supporting its therapeutic value in nerve repair, functional restoration, pain management, and treatment of degenerative neurological conditions [8, 31–36]. The comprehensive body of evidence regarding PRP applications in various peripheral nerve regeneration scenarios is summarized in Figure 1, providing a systematic overview of treatment protocols, outcomes, and clinical effectiveness across different nerve injury types and anatomical locations.

### Cellular and molecular mechanisms driving PRP therapy effects

The process of concentrating and separating platelets from the patient’s own blood is known as PRP therapy. Upon activation, these platelets release a potent array of bioactive molecules stored within their granules, including cytokines, growth factors, and signaling molecules. These components are fundamental orchestrators of tissue repair and wound healing processes [37]. A primary mechanism involves the direct stimulation of cellular proliferation and differentiation essential for regeneration. Growth factors within PRP, such as PDGF, TGF-β, and VEGF, activate key cell types: mesenchymal stem cells (MSCs), promoting their proliferation and differentiation into various tissue-specific lineages; endothelial cells, stimulating angiogenesis to improve local vascularization; and fibroblasts, enhancing their synthesis of crucial extracellular matrix (ECM) components like collagen and fibronectin [38, 39].

Furthermore, PRP actively promotes the production of essential structural molecules, including fibronectin, collagen, and hyaluronic acid. Collectively, these molecules form a provisional ECM scaffold. This scaffold provides critical mechanical support to the healing site, facilitates cell migration, and creates a conducive environment for tissue regeneration [40]. Critically, PRP also activates and recruits local endogenous stem cells within the injury site, amplifying their potential to differentiate into the specific cell types needed for functional tissue repair [41]. Crucially, the efficacy of these regenerative processes, cellular activation, differentiation, and ECM synthesis, is profoundly enhanced by PRP’s ability to modulate the inflammatory environment, shifting it towards a state optimal for repair [42, 43]. PRP exerts significant immunomodulatory actions, suppressing detrimental chronic inflammation and actively promoting resolution and regeneration. This anti-inflammatory activity is intrinsically linked to creating the permissive conditions necessary for the cellular and matrix-building activities described previously.

PRP achieves this essential immunomodulation through several interconnected pathways. Firstly, it serves as a rich source of potent anti-inflammatory molecules, including interleukin-1 receptor antagonist (IL-1ra), Interleukin-4 (IL-4), and Interleukin-10 (IL-10). IL-1ra directly inhibits the potent pro-inflammatory cytokine IL-1β, while IL-4 and IL-10 suppress the production of other key pro-inflammatory mediators like IL-6 and TNF-α, simultaneously promoting anti-inflammatory signaling cascades [42, 44]. Secondly, PRP stimulates the polarization of macrophages away from the pro-inflammatory M1 phenotype towards the anti-inflammatory, pro-repair M2 phenotype. These M2 macrophages secrete high levels of TGF-β and IL-10, further dampening inflammation, exhibit enhanced phagocytic activity to clear cellular debris, and directly contribute to tissue remodeling [44]. Thirdly, components within PRP, notably TGF-β and Prostaglandin E2 (PGE2), act to regulate T-cell responses. TGF-β induces cell cycle arrest and apoptosis in T-cells, while PGE2 downregulates essential co-stimulatory molecules and cytokine receptors on their surface, thereby inhibiting T-cell activation and proliferation [45]. Fourthly, PRP influences dendritic cell (DC) function, promoting the development of tolerogenic DCs. These specialized DCs exhibit reduced expression of pro-inflammatory cytokines and co-stimulatory molecules, instead fostering immune tolerance and the generation of regulatory T-cells (Tregs) [46]. Finally, PRP directly enhances the development, proliferation, and function of Tregs themselves. Tregs are essential for maintaining immune tolerance; they suppress effector T-cells and other immune cells through mechanisms involving the release of anti-inflammatory cytokines (IL-10, TGF-β) and direct cell contact [47].

**FIGURE 2:**
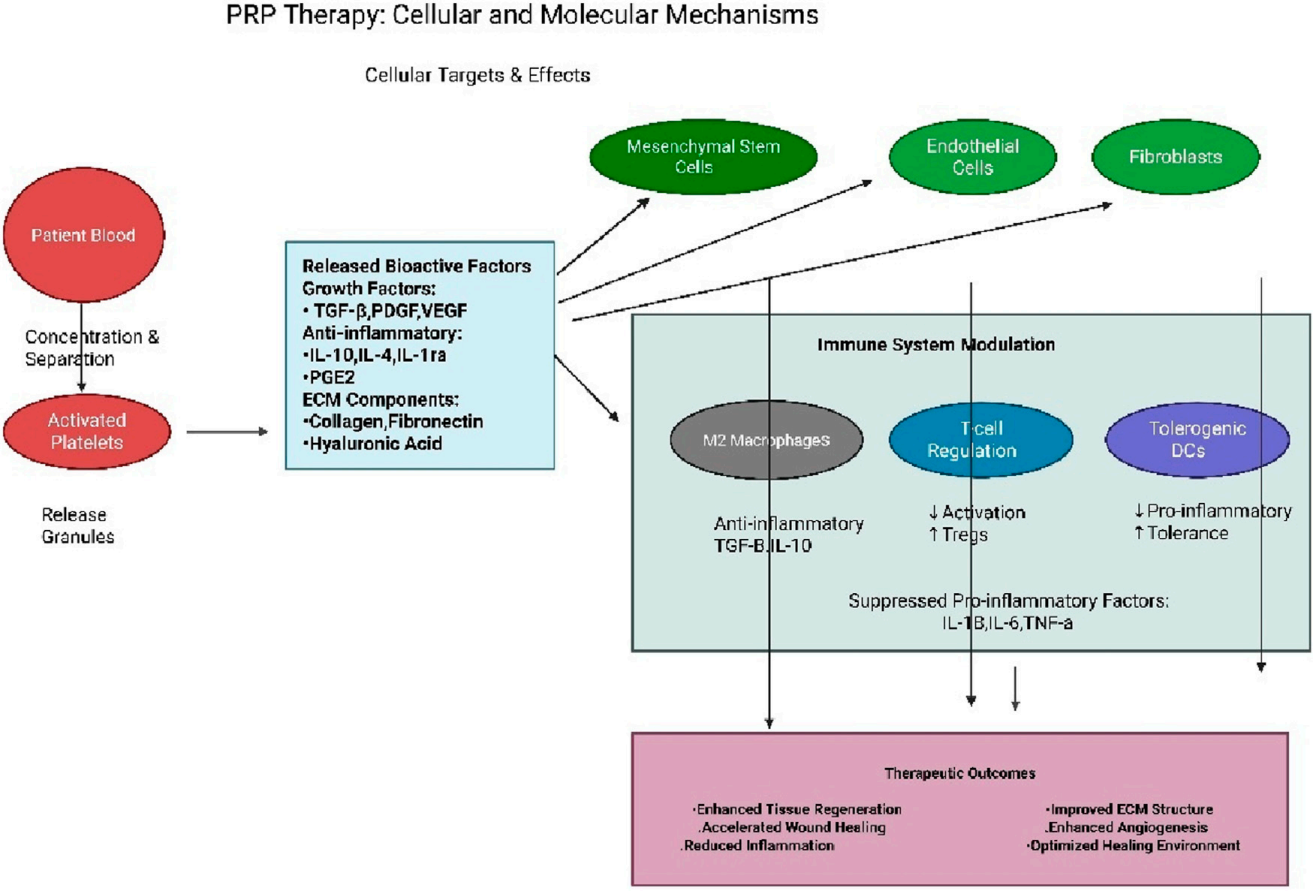
The schematic presentation of PRP cellular and molecular therapy.

By orchestrating this complex immunomodulation alongside its direct regenerative effects on cells and matrix synthesis, PRP comprehensively supports the sequential phases of wound healing. During the initial inflammatory phase, PRP helps resolve inflammation efficiently and promotes the formation of granulation tissue. Subsequently, it actively drives the proliferation and migration of key cells like fibroblasts, endothelial cells, and keratinocytes. Finally, it supports the remodeling phase by providing the necessary matrix components and signals. This integrated action accelerates the overall healing process and enhances the functional quality and structural integrity of the regenerated tissue [48]. A schematic overview of PRP’s cellular and molecular mechanisms is presented in Figure 2.

### The combination of surgical and rehabilitative techniques with PRP therapy

The combination of surgical and rehabilitative techniques with PRP therapy encompasses multiple temporal approaches, each serving distinct therapeutic purposes. A recent exploratory study in rabbits demonstrated that preoperative PRP treatment of the implantation site significantly enhanced fat graft survival, with decreased inflammation and fibrosis and markedly improved angiogenesis compared to control groups [49].

Building upon this preoperative foundation, the therapeutic potential of PRP can be further maximized through its strategic application during the surgical procedure itself. Intraoperative PRP application refers to the planned incorporation of PRP as an integral component of the surgical procedure. This approach is typically applied during the final stages of surgery rather than throughout the entire operation, representing a primary therapeutic goal rather than an ancillary treatment. Surgeons strategically apply platelet-rich plasma directly to the surgical site or incorporate it into biological scaffolds immediately before wound closure. This optimizes tissue integration and regeneration through growth factors and bioactive substances that stimulate neovascularization and extracellular matrix production, ensuring maximum therapeutic benefit while the surgical site remains accessible [50].

Postoperative PRP therapy involves administering PRP injections into the surgical site during the recovery period to accelerate tissue regeneration and enhance functional recovery. This approach differs from intraoperative application as it occurs days to weeks after the initial surgical procedure. Postoperative PRP significantly accelerates tissue regeneration, reduces post-surgical inflammation, and promotes optimal wound healing during the critical recovery phase. The therapy directly delivers growth factors and cytokines to the surgical site through targeted injections that promote angiogenesis, collagen deposition, and cellular proliferation [51].

Integration with rehabilitation protocols represents an advanced approach where PRP therapy is strategically combined with physical therapy and rehabilitation programs following surgical procedures. Physical therapists can incorporate PRP injections as a complementary tool within comprehensive rehabilitation programs to enhance neuromuscular re-education, improve joint stability, and accelerate tissue repair processes. This integrated approach leverages PRP’s ability to enhance the healing of injured tissues, reduce pain levels, and improve muscle strength. The synergistic effect of combining biological enhancement with mechanical stimulation ultimately enables a more complete and expedited return to pre-injury functional capacity [52].

### Impact of PRP in peripheral nerve injury recovery

The role of PRP in peripheral nerve injury recovery has been summarized in Table 1. PRP represents a concentrated autologous preparation derived from patient blood that contains elevated concentrations of platelets and bioactive growth factors essential for tissue regeneration. The therapeutic mechanism underlying PRP’s efficacy centers on its ability to secrete critical growth factors, including PDGF, which promotes cellular proliferation; TGF-β, which modulates inflammatory responses and stimulates tissue repair; and VEGF, which promotes neovascularization to support regenerating neural tissue. Given that PRP is autologous in nature, it exhibits minimal risk of immunogenic reactions, establishing it as a promising therapeutic modality for peripheral nerve injury management [76].

**TABLE 1:** Therapeutic potential of PRP in peripheral nerve injury treatment.

PRP treatment	Outcomes in PNI	Species	References
PRP	PRP enhances locomotor recovery, spares white matter, promotes angiogenesis and neuronal regeneration, and modulates blood vessel size, leading to the recovery of spinal cord injuries	Rat	[15]
PRP	Promoted the radial nerve in musculoskeletal disorders	Human	[20]
PRP-derived exosomes	Promoted SC proliferation and dorsal root ganglion axon growth Increased the ability of MSCs to promote neural repair and regeneration in patients with PNI.	Mouse	[23, 24]
PRP gel +Collagen film	Promoted facial nerve regeneration	Rats	[53]
PRP	Recovered facial nerve injury followed by promoting vibrissae movement, eyelid closure, and electrophysiological function	Rat	[54]
PRP gel +Collagen/Chitosan composite film	Promoted the proliferation of SC, nerve regeneration and functional recovery in rats with sciatic nerve injury	Rat	[55]
PRP+ Low-dose ultrashort wave therapy	Improve sciatic nerve crush injury regeneration and recovery	Rabbit	[30]
PRP	Promoted nerve regeneration through improvement of angiogenesis and intracellular ubiquitin levels by regulating ITGB8, ribosomal protein S27a (RSP27a) and ubiquilin 1 (UBQLN1)	Rabbit	[56]
PRP + gelatin methacrylate and sodium alginate hydrogel	Enhances growth factors VEGF-A and PDGF-followed by promotion of the migration of SC and the neovascularization of endothelial cells to prevent sciatic nerve defects and facilitate the repairing of peripheral nerve	Rat	[57]
Platelet-rich fibrin	The axon regeneration of the sciatic nerve and sensory function was improved with nerve conduit filled with platelet-rich fibrin Repair peripheral nerve defects	Mouse	[58]
PRP-derived exosomes	Promoted the nerve regeneration by enhancing the proliferation, migration, and secretion of trophic factors by SC	Rat	[59]
PRP	Enhanced the proliferation, secretion and migration of SCs and the regeneration of axons in the early stage as well as VEGF expression and improved repairing of tibial nerve defects	Rabbit	[60]
Freeze-dried PRP	Increased the expression of nerve growth factor and S100B Induced neuro-regeneration and relieved chronic orofacial pain	Rats	[61]
PRP	Treatment of recurrent laryngeal nerve injury Regeneration Promoted the proliferation and migration of SC	Rabbit	[62]
Human umbilical cord blood+ PRP	Improved the spinal cord injury regeneration	Rat	[63, 64]
PRP + chitin	Facilitate the repairing of sciatic nerve defects	Rat	[65]
Platelet-rich fibrin	Enhanced the sciatic nerve regeneration	Rat	[66]
PRP	Enhanced the mature SC proliferation, and microenvironment in the small gap and promote peripheral nerve regeneration	Rabbit	[67]
PRP+ adipose tissue–derived stem cells	Improve recovering of sciatic nerve repairing and prevent its defects	Rat	[68]
PRP+ adipose tissue–derived stem cells	Enhanced the spinal cord injury recovery and improved of repairing central nervous system	Rat	[69]
PRP + Citicoline	Improved sciatic nerve injury following recovery of peripheral nerve injury	Rat	[70]
Leukocyte-platelet rich fibrin	Suppressed proinflammatory cytokines followed by prevention of peripheral nerve inflammation and injuries Facilitated peripheral nerve regeneration	Rat	[71]
PRP	Induces nerve regeneration by promoting neurotrophic factors and anti-inflammatory cytokines by calcium chloride activation Facilitated the recovery of spinal cord dorsal root repair	Rat	[71]
PRP	Improved regeneration and proliferation of SC	Rabbit	[72]
Platelet-rich fibrin	Facilitated the regeneration of sciatic nerves and peripheral nerve injury	Rat	[73]
Platelet-rich fibrin	Improved the regeneration of sciatic nerve Showed positive effect on maxillofacial tissues regeneration	Rabbit	[74]
PRP	Promoted the healing of digital nerve crush injury Decreased the neuropathic pain	Human	[75]

Numerous preclinical and clinical studies across various nerve types, including facial, sciatic, and median nerves, have widely supported the positive effect of PRP on peripheral nerve healing [30, 53, 55–57, 77–85]. In models of facial nerve injury, experimental research showed that PRP can greatly enhance therapeutic outcomes when combined with biocompatible materials like chitosan, which serves as a structured scaffold for controlled and sustained release of growth factors at the injury site [53, 78]. Li et al. showed that PRP has neuroprotective effects on traumatic facial nerve injuries, with notable recovery of SC and significant axonal regeneration [54]. Likewise, studies on sciatic nerve injury consistently indicate that autologous PRP supports nerve regeneration by decreasing M1 macrophages and altering the inflammatory environment [32, 79, 80, 86–89].

PRP has been shown to stimulate SC proliferation and secretion while promoting angiogenesis and affecting intracellular signaling pathways. Notably, PRP significantly upregulated the expression of integrin subunit β-8 (ITGB8), which plays a critical role in angiogenesis after nerve injury [56]. When combined with biomaterial scaffolds such as collagen/chitosan composite membranes or gelatin methacrylate hydrogels, PRP has demonstrated enhanced efficacy in promoting both functional and structural nerve recovery [55, 81, 82].

In median nerve applications, particularly for carpal tunnel syndrome treatment, PRP has shown superior outcomes compared to corticosteroids in clinical trials, providing significant pain relief and functional improvement [77, 83–85, 90–93]. Studies have demonstrated that ultrasound guided PRP injections can provide effective treatment for up to 1 year post-injection, with predictive factors including patient body weight, distal motor latency, and median nerve cross-sectional area [77, 85].

In recent years, progress in platelet research has highlighted the significance of platelet-derived extracellular vesicles (EVs), including exosomes, and their role in facilitating intercellular communication [94]. A study conducted by Yi et al. [59] isolated platelet-rich plasma–derived exosomes (PRP-Exos) and found that they markedly promoted SC proliferation, migration, and secretion of trophic factors. Additionally, PRP-Exos induced notable changes in both transcriptional and protein expression within SCs, especially increasing the expression of genes crucial for nerve repair. In a rat sciatic nerve crush model, the application of ultrasound-targeted microbubble destruction (UTMD) significantly improved the delivery of PRP-Exos to the injury site, resulting in greater exosome accumulation locally and enhanced regenerative and functional outcomes compared to untreated controls [59].

Other studies have shown that PRP-Exos improve MSC survival by reducing apoptosis, preserving stemness, and delaying senescence. Pretreated MSCs (pExo-MSCs) demonstrated better retention *in vivo*, resulting in enhanced axonal regeneration, remyelination, and neurological recovery. *In vitro*, they further encouraged SC proliferation and dorsal root ganglion axonal extension, mainly through glial cell–derived neurotrophic factor (GDNF) secretion and activation of the PI3K/Akt pathway [24].

Similarly, Zhang et al. (2024) reported that PRP-Exo–treated MSCs (MSC^PExo) enhanced SC proliferation and reduced apoptosis after peripheral nerve injury (PNI). Conditioned medium from MSC^PExo^ (MSC^PExo-CM^) further stimulated SC proliferation, migration, and angiogenesis. Proteomic analysis of the MSC^PExo^ secretome identified 440 proteins, many of which showed strong pro-regenerative and angiogenic functions. ELISA confirmed the enrichment of key trophic factors, and Western blotting validated PI3K/Akt pathway activation. Collectively, these findings highlight PRP-Exos as potent enhancers of MSC paracrine activity and valuable modulators of neural repair [23].

## Factors affecting PRP therapy in nerve repair

**FIGURE 3:**
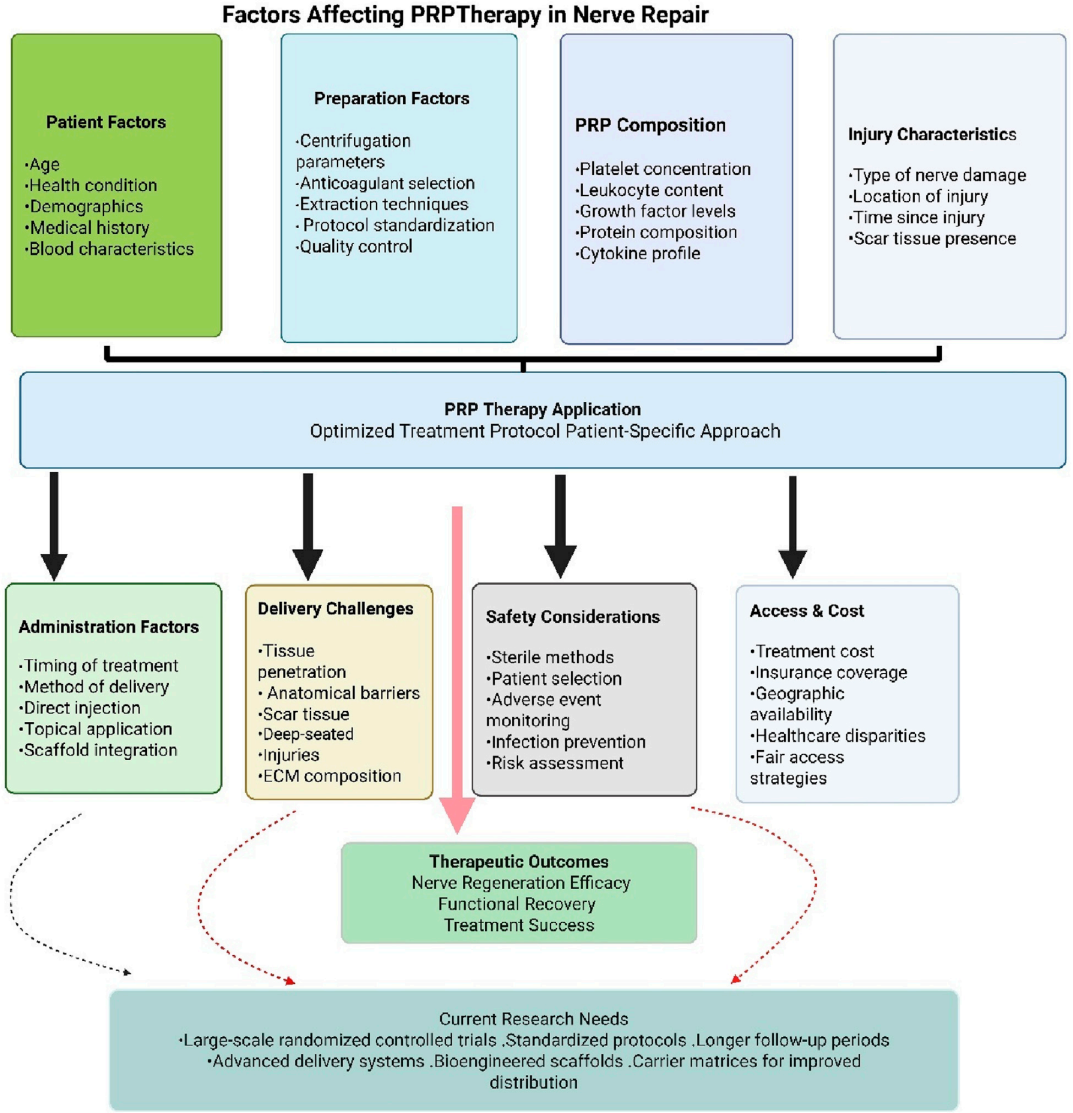
Summary of factors affecting PRP therapeutic potential.

The factors affecting PRP effectiveness are detailed in Table 2 and depicted graphically in Figure 3 for enhanced clarity. The technique used for preparation, the parameters of centrifugation, and patient-specific characteristics such as age and health condition can significantly influence the composition of PRP. Research demonstrates that PRP efficacy decreases with increasing age, with PRP derived from young donors (18–35 years) showing significantly better therapeutic outcomes compared to PRP from older donors (≥65 years) [124]. Studies show that growth factor levels, including PDGF-BB, TGF-β1, IGF-1, and EGF, are statistically higher in subjects younger than 25 years compared to those aged 26 years or older [125]. Additionally, PRP derived from women older than 45 years does not contain significantly higher concentrations of bioactive components compared to younger groups, suggesting that aging significantly affects the active components of PRP [126]. At the cellular level, elderly patients show decreased numbers of α-granules in platelets, which are the main component releasing active substances, leading to decreased platelet function [127]. Clinical evidence supports these laboratory findings, with PRP therapy showing poor efficacy in elderly patients (≥60 years) for conditions such as facial rejuvenation and Achilles tendinitis treatment [124].

**TABLE 2:** Factors affecting the efficacy of PRP.

Parameters affecting PRP efficacy	Biological outcomes of PRP	References
Concentration of platelet	The platelets concentration in PRP can vary depending on how it is prepared, and the equipment used. Higher platelet concentrations are generally associated with better outcomes, but there is an optimal range, and too high concentrations may not be beneficial	[95, 96]
Contents of leukocyte	PRP can be categorized as either leukocyte-rich or leukocyte-poor, depending on whether leukocytes are present or absent. The amount of leukocytes present can impact the inflammatory response and the healing of tissues	[97–100]
Method of activation for PRP	PRP can be activated through different methods, including thrombin, calcium chloride, or exposure to collagen. This activation subsequently triggers the release of growth factors from platelets, thereby influencing the regenerative properties of PRP.	[101–104]
Buffy coat removal	The method used to separate the buffy coat from whole blood during PRP preparation determines the purity and composition of PRP.	[105, 106]
Time and speed utilized for centrifugation	The separation of blood components and the final composition of PRP are determined by the speed and duration of centrifugation. It is crucial to use optimal centrifugation parameters to obtain PRP with the desired properties	[107–109]
Types of anticoagulants	Anticoagulants like citrate or heparin are utilized to prevent clotting while collecting blood. The selection of anticoagulant can impact the activation of platelets and the stability of PRP.	[43, 106, 110]
Injectable formulation	PRP can be administered in either liquid or gel form, depending on the specific clinical application. The injectable form chosen has a significant impact on the ease of administration and how PRP is distributed within the tissues	[111–114]
Composition of growth factor	The concentration and composition of growth factors, such as TGF-β, PDGF, and VEGF, can vary among different PRP preparations. The specific growth factors released by the platelets and their concentrations play a critical role in the regenerative and healing processes	[115–117]
Injected PRP volume	The distribution, diffusion, and therapeutic effects of PRP in the target tissue can be influenced by the volume injected	[70, 107]
Factors specific to the patient	The response to PRP treatment can be affected by various factors, including age, sex, underlying health conditions, and medications	[118–120]
Clinical hallmarks	The choice of PRP preparation and administration protocol is influenced by the specific condition being treated, such as tendonitis, osteoarthritis, or wound healing, as well as the targeted tissue PRP therapy may be more effective for certain types of tissues, such as tendons, ligaments, and cartilage, compared to others. Additionally, mild to moderate injuries tend to respond better to PRP than severe or chronic conditions. Furthermore, areas with a good blood supply may exhibit enhanced healing with the use of PRP therapy	[18, 34, 121]
Content of fibrin	Fibrin, present in PRP, plays a crucial role in both clot formation and tissue healing. Certain classification systems differentiate PRP preparations based on their fibrin content, categorizing them as either fibrin-rich or fibrin-poor, according to their clotting characteristics and regenerative capabilities	[43, 122]
Contamination of red blood cells	Contamination of red blood cells (RBCs) in PRP can significantly impact the quality and efficacy of PRP in various therapeutic applications	[41, 123]

The therapeutic efficacy of PRP in peripheral nerve repair demonstrates significant variability due to differences in leukocyte content, growth factor concentrations, and platelet density across various PRP formulations [128–130]. This heterogeneity underscores the critical need for standardized preparation protocols encompassing appropriate anticoagulant selection, optimal centrifugation parameters, and consistent extraction methodologies to ensure reproducible outcomes in both research and clinical applications [131, 132]. Despite encouraging results from preclinical investigations and early-phase clinical trials, the current body of clinical evidence remains insufficient to definitively establish PRP’s effectiveness in nerve repair and regeneration. Existing studies frequently exhibit methodological limitations, including inadequate sample sizes, absence of appropriate control groups, heterogeneous patient populations, and insufficient follow-up durations. Large-scale randomized controlled trials employing standardized protocols and extended observation periods are essential to establish the safety profile, therapeutic efficacy, and optimal clinical applications of PRP therapy in nerve regeneration [133]. Achieving adequate PRP distribution and tissue penetration presents additional complexities, particularly in cases involving scar tissue formation or deep-seated injuries. Anatomical barriers, tissue density variations, and extracellular matrix composition may impede PRP penetration into the neuronal microenvironment, potentially limiting regenerative efficacy. Advanced delivery systems incorporating carrier matrices or bioengineered scaffolds may enhance PRP distribution and retention at nerve injury sites [88]. While PRP therapy generally demonstrates a favorable safety profile, specific risks associated with nerve regeneration applications include hypersensitivity reactions, iatrogenic nerve injury, hematoma formation, and infection. Rigorous adherence to sterile protocols, careful patient selection criteria, and comprehensive adverse event monitoring are essential to minimize these risks and ensure treatment safety [134]. Additionally, therapeutic accessibility remains constrained by economic factors, particularly in regions where insurance coverage or healthcare systems do not support the costs of PRP therapy. Geographic and institutional limitations further compound healthcare disparities. Addressing these challenges requires comprehensive strategies to reduce treatment costs, expand reimbursement coverage, and improve therapeutic accessibility to ensure equitable patient access to this potentially beneficial regenerative approach [110].

## Conclusion

The exploration of PRP as a treatment target for PNIs has shown significant promise, especially when PRP is obtained through plasmapheresis. This review emphasizes the positive outcomes seen in both clinical and preclinical studies, where PRP treatment has been linked to better nerve regeneration, improved sensory and motor functions, and less pain. Preclinical studies have provided valuable insights into how PRP promotes nerve repair, including encouraging axonal growth and reducing scar formation. Despite these promising results, several obstacles remain when turning preclinical findings into clinical practice. These include species-specific differences and the need for thorough clinical evaluations to confirm safety and effectiveness in humans. Standardizing PRP preparation methods and optimizing treatment timing are essential steps to improve the consistency and reliability of PRP therapy outcomes. Future research should aim to better understand the molecular mechanisms behind PRP’s therapeutic effects, refine treatment protocols, and expand its clinical use. By tackling these challenges and integrating insights from both human and animal studies, the full potential of PRP as a strong option for nerve regeneration and functional recovery in patients with PNI can be achieved.
